# Di-μ-acetato-μ-aqua-bis­[acetatobis(1*H*-benzimidazole)cobalt(II)]

**DOI:** 10.1107/S1600536808015596

**Published:** 2008-05-30

**Authors:** Iwan Zimmermann, Tony D. Keene, Antonia Neels, Silvio Decurtins

**Affiliations:** a Departement für Chemie und Biochemie, Universität Bern, Freiestrasse 3, CH-3012 Bern, Switzerland; b Institut de Microtechnique, Jaquet Droz 1, CP 526, CH-2002 Neuchâtel, Switzerland

## Abstract

In the title compound, [Co_2_(C_2_H_3_O_2_)_4_(C_7_H_6_N_2_)_4_(H_2_O)], the half-mol­ecule in the asymmetric unit is completed by a crystallographic twofold rotation axis to give the full mol­ecule. The Co^II^ ions are approximately octahedrally coordinated with a *cis*-N_2_O_4_ coordination sphere. The compound features intra­molecular O—H⋯O hydrogen bonds between the non-bridging acetate groups and the bridging water mol­ecule, and inter­molecular N—H⋯O hydrogen bonds between the acetates and amine H atoms of the benzimidazoles which determine the mol­ecular packing in the crystal structure.

## Related literature

For related literature, see: Brown *et al.* (2004 ▶); Hagen *et al.* (1993 ▶); Orpen *et al.* (1989 ▶); Turpeinen *et al.* (1987 ▶); Ye *et al.* (1997 ▶).
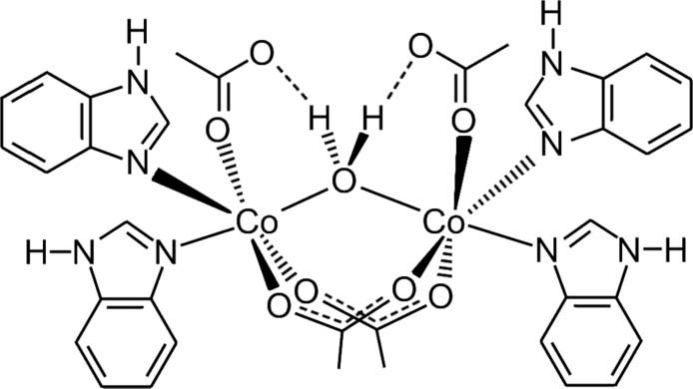


## Experimental

### 

#### Crystal data

[Co_2_(C_2_H_3_O_2_)_4_(C_7_H_6_N_2_)_4_(H_2_O)]
*M* *_r_* = 844.6
Orthorhombic, 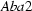

*a* = 18.663 (4) Å
*b* = 8.8101 (18) Å
*c* = 22.727 (5) Å
*V* = 3736.7 (13) Å^3^
*Z* = 4
Mo *K*α radiation
μ = 0.95 mm^−1^
*T* = 150 (2) K
0.1 × 0.09 × 0.08 mm

#### Data collection

Stoe IPDS diffractometer
Absorption correction: multi-scan (*MULscanABS* in *PLATON*; Spek, 2003 ▶) *T*_min_ = 0.914, *T*_max_ = 0.956
24157 measured reflections
5048 independent reflections
3698 reflections with *I* > 2σ(*I*)
*R*_int_ = 0.069

#### Refinement

*R*[*F* ^2^ > 2σ(*F* ^2^)] = 0.035
*wR*(*F* ^2^) = 0.077
*S* = 0.89
5048 reflections
252 parameters
1 restraint
H-atom parameters constrained
Δρ_max_ = 0.36 e Å^−3^
Δρ_min_ = −0.57 e Å^−3^
Absolute structure: Flack (1983 ▶), with 2433 Friedel pairs
Flack parameter: 0.006 (12)

### 

Data collection: *EXPOSE* in *IPDS Software* (Stoe & Cie, 2000 ▶); cell refinement: *CELL* in *IPDS Software*; data reduction: *INTEGRATE* in *IPDS Software*; program(s) used to solve structure: *SIR92* (Altomare *et al.*, 1994 ▶); program(s) used to refine structure: *SHELXL97* (Sheldrick, 2008 ▶); molecular graphics: *DIAMOND* (Brandenberg, 1999 ▶); software used to prepare material for publication: *SHELXL97* and *WinGX* (Farrugia, 1999 ▶).

## Supplementary Material

Crystal structure: contains datablocks global, I. DOI: 10.1107/S1600536808015596/si2092sup1.cif

Structure factors: contains datablocks I. DOI: 10.1107/S1600536808015596/si2092Isup2.hkl

Additional supplementary materials:  crystallographic information; 3D view; checkCIF report

## Figures and Tables

**Table:** 

Co1—O3	2.0495 (18)
Co1—O4^i^	2.1044 (19)
Co1—O1	2.1141 (17)
Co1—O1*W*	2.1315 (15)
Co1—N1	2.139 (2)
Co1—N3	2.147 (2)

**Table:** 

O3—Co1—O4^i^	97.48 (8)
O3—Co1—O1	174.69 (7)
O4^i^—Co1—O1	86.88 (7)
O3—Co1—O1*W*	90.09 (6)
O4^i^—Co1—O1*W*	90.78 (7)
O1—Co1—O1*W*	92.89 (6)
O3—Co1—N1	88.66 (8)
O4^i^—Co1—N1	87.79 (8)
O1—Co1—N1	88.48 (7)
O1*W*—Co1—N1	177.97 (9)
O3—Co1—N3	88.14 (8)
O4^i^—Co1—N3	174.38 (8)
O1—Co1—N3	87.50 (8)
O1*W*—Co1—N3	89.38 (8)
N1—Co1—N3	92.19 (8)
Co1^i^—O1*W*—Co1	116.98 (13)

Symmetry code: (i) 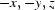.

**Table 2:** Hydrogen-bond geometry (Å, °)

*D*—H⋯*A*	*D*—H	H⋯*A*	*D*⋯*A*	*D*—H⋯*A*
O1*W*—H1*W*⋯O2	0.97	1.71	2.591 (2)	149
N4—H22⋯O2^ii^	0.86	1.87	2.717 (3)	167
N2—H12⋯O4^iii^	0.86	2.00	2.812 (2)	157

Symmetry codes: (ii) 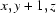; (iii) 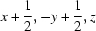.

**Fig. 1.**
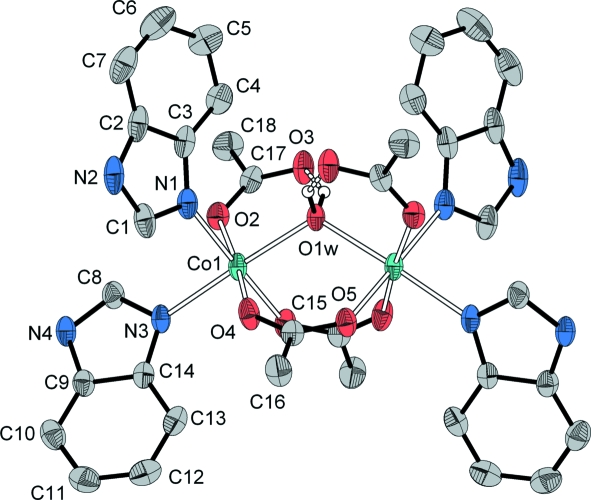
Asymmetric unit and selected symmetry equivalents with thermal ellipsoids at the 50% probability level. Hydrogen atoms (except H1w) and atom labels for the symmetry equivalent atoms (symmetry code: -x, -y, z) omitted for clarity. Hydrogen bonds depicted by dashed lines.

**Fig. 2.**
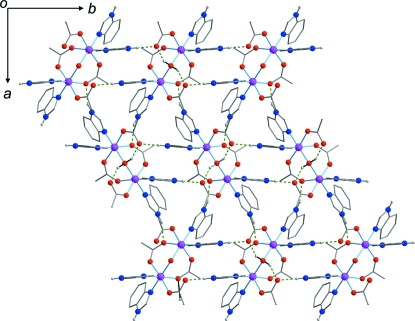
View of the hydrogen bonding network (dashed green bonds) and crystal packing, looking down the c-axis.

## supplementary crystallographic information

### Comment

The title compound belongs to an extensive group of compounds which share the same (µ_2_-acetato)-(µ_2_-aquo)-di-*M*(II) core (see for example Turpeinen *et al.* 1987; Brown *et al.* 2004; Hagen *et al.* 1993), indicating that this motif is favourable under a variety of synthetic conditions and for many different metals. The compound crystallizes from a solution of benzimidazole, cobalt acetate tetrahydrate and oxalic acid dihydrate in methanol which was left to evaporate over five days at room temperature. The title complex, with its bridging water molecule lying on a twofold rotation axis, is isostructural with the previously reported Mn(II) congener (Ye *et al.*, 1997) and the structure comprises two Co(II) ions bridged by two acetate anions and one water molecule with the remainder of each approximately octahedral Co(II) coordination sphere being completed by coordination by the imine N atoms from two benzimidazole molecules and one monodentate acetate group (Fig. 1). All bond lengths and angles are in accordance with literature values (Orpen *et al.*, 1992). The water molecule hydrogen bonds to the uncoordinated oxygen atom of the monodentate acetate groups within the dimer with a short H···O distance of 1.714 (2) Å (Fig. 2, Table 2). The monodentate acetate groups show that there is a large degree of delocalization in the carboxylate group with C—O bonds of 1.250 (3) (C1—O1) and 1.269 (3) Å (C1—O2), (Table 1). The shorter C1—O1 distance implies that this bond has more of a carbonyl character than C1—O2, as seen in the Mn(II) congener, indicating that the hydrogen bond between O2 and H1w affects the delocalization. The amine hydrogen atoms all take part in hydrogen bonds to acetate anions in neighbouring dimer groups to create the crystal packing. All acetates are involved in intermolecular hydrogen bonding, which forms planes of dimers in the *ab*-plane (Fig. 2, Table 2).

### Experimental

Single crystals of the title compound suitable for X-ray diffraction experiments were obtained by dissolving cobalt(II) acetate tetrahydrate (1 mmol, 249 mg) and oxalic acid dihydrate (1 mmol, 126 mg) in a saturated solution of benzimidazole in methanol (30 ml). The subsequent solution was left to evaporate at room temperature for five days before red crystals formed. IR (KBr disc, transmission, cm^-1^): 3088 (*m*), 2982 (*m*), 2910 (*m*), 2836 (w), 1629 (*s*), 1606 (*s*), 1491 (*m*), 1420 (*s*), 1341 (w), 1304 (*m*), 1273 (*m*), 1251 (*m*), 1011 (w), 963 (w), 887 (w), 775 (w), 741 (*s*), 653 (*m*), 619 (w), 547 (w), 428 (w).

### Refinement

All hydrogen atoms were fixed in calculated positions and refined in riding mode with *U*_iso_(H) = 1.2 *U*_eq_. O—H, N—H and C—H bond lengths were fixed to 0.97, 0.86 and 0.96 Å, respectively. The number of Friedel pairs was 2433.

### Crystal data

**Table:**

[Co_2_(C_2_H_3_O_2_)_4_(C_7_H_6_N_2_)_4_(H_2_O)]	*D*_x_ = 1.501 Mg m^−^^3^
*M**_r_* = 844.6	Melting point: N/A K
Orthorhombic, *A**b**a*2	Mo *K*α radiation λ = 0.71073 Å
Hall symbol: A 2 -2ac	Cell parameters from 15135 reflections
*a* = 18.663 (4) Å	θ = 1.8–29.5º
*b* = 8.8101 (18) Å	µ = 0.95 mm^−^^1^
*c* = 22.727 (5) Å	*T* = 150 (2) K
*V* = 3736.7 (13) Å^3^	Block, red
*Z* = 4	0.1 × 0.09 × 0.08 mm
*F*_000_ = 1744	

### Data collection

**Table:**

Stoe IPDS diffractometer	*R*_int_ = 0.069
φ oscillation scans	θ_max_ = 29.3º
Absorption correction: multi-scan(MULscanABS in PLATON03; Spek, 2003)	θ_min_ = 1.8º
*T*_min_ = 0.914, *T*_max_ = 0.956	*h* = −25→25
24157 measured reflections	*k* = −12→11
5048 independent reflections	*l* = −31→31
3698 reflections with *I* > 2σ(*I*)	

### Refinement

**Table:**

Refinement on *F*^2^	*w* = 1/[σ^2^(*F*_o_^2^) + (0.0441*P*)^2^] where *P* = (*F*_o_^2^ + 2*F*_c_^2^)/3
Least-squares matrix: full	(Δ/σ)_max_ < 0.001
*R*[*F*^2^ > 2σ(*F*^2^)] = 0.035	Δρ_max_ = 0.36 e Å^−^^3^
*wR*(*F*^2^) = 0.078	Δρ_min_ = −0.57 e Å^−^^3^
*S* = 0.89	Extinction correction: SHELXL97 (Sheldrick, 2008), Fc^*^=kFc[1+0.001xFc^2^λ^3^/sin(2θ)]^-1/4^
5048 reflections	Extinction coefficient: 0.00058 (8)
252 parameters	Absolute structure: Flack (1983), 2433 Friedel pairs
1 restraint	Flack parameter: 0.006 (12)
H-atom parameters constrained	

### Special details

**Table:**

Geometry. All e.s.d.'s (except the e.s.d. in the dihedral angle between two l.s. planes) are estimated using the full covariance matrix. The cell e.s.d.'s are taken into account individually in the estimation of e.s.d.'s in distances, angles and torsion angles; correlations between e.s.d.'s in cell parameters are only used when they are defined by crystal symmetry. An approximate (isotropic) treatment of cell e.s.d.'s is used for estimating e.s.d.'s involving l.s. planes.

### Fractional atomic coordinates and isotropic or equivalent isotropic displacement parameters (Å^2^)

**Table:**

	*x*	*y*	*z*	*U*_iso_*/*U*_eq_	
Co1	0.081808 (12)	0.11188 (3)	0.166836 (17)	0.02569 (8)	
O1W	0	0	0.21585 (12)	0.0248 (5)	
H1W	0.0227	−0.0745	0.2412	0.03*	
O2	0.10002 (8)	−0.1571 (2)	0.26630 (9)	0.0362 (4)	
O1	0.16471 (8)	0.0148 (2)	0.21784 (9)	0.0305 (4)	
O4	−0.09188 (8)	0.0772 (2)	0.11088 (9)	0.0314 (4)	
O3	0.00530 (9)	0.2253 (2)	0.11937 (9)	0.0365 (4)	
N1	0.16209 (10)	0.2216 (2)	0.11482 (10)	0.0300 (5)	
N4	0.08897 (11)	0.5364 (3)	0.25660 (14)	0.0434 (6)	
H22	0.0924	0.6335	0.2537	0.052*	
N3	0.07962 (10)	0.2950 (2)	0.22915 (11)	0.0318 (5)	
N2	0.26092 (9)	0.3580 (3)	0.10253 (11)	0.0346 (5)	
H12	0.3015	0.3996	0.1104	0.042*	
C27	0.08124 (12)	0.3017 (3)	0.29057 (13)	0.0331 (6)	
C26	0.07634 (14)	0.1894 (3)	0.33343 (13)	0.0389 (6)	
H26	0.0717	0.0877	0.3232	0.047*	
C17	0.16144 (11)	0.2838 (3)	0.05885 (12)	0.0268 (5)	
C22	0.08784 (13)	0.4537 (3)	0.30791 (15)	0.0386 (7)	
C12	0.22350 (12)	0.3704 (3)	0.05085 (12)	0.0298 (5)	
C21	0.08369 (13)	0.4376 (3)	0.21186 (15)	0.0390 (6)	
H21	0.083	0.4669	0.1725	0.047*	
C11	0.22226 (12)	0.2688 (3)	0.13860 (12)	0.0329 (6)	
H11	0.2367	0.2431	0.1765	0.04*	
C3	−0.05615 (12)	0.1965 (3)	0.10030 (12)	0.0286 (5)	
C13	0.23834 (15)	0.4491 (3)	−0.00066 (13)	0.0409 (7)	
H13	0.2797	0.5071	−0.0049	0.049*	
C16	0.11186 (14)	0.2719 (3)	0.01321 (13)	0.0350 (6)	
H16	0.0706	0.2136	0.0173	0.042*	
C2	0.22415 (14)	−0.1402 (4)	0.28792 (14)	0.0439 (7)	
H2A	0.2663	−0.1147	0.2658	0.066*	
H2B	0.2225	−0.2479	0.294	0.066*	
H2C	0.2255	−0.0895	0.3253	0.066*	
C1	0.15851 (12)	−0.0905 (3)	0.25437 (12)	0.0286 (5)	
C4	−0.09095 (15)	0.3150 (4)	0.06247 (15)	0.0434 (7)	
H4A	−0.1143	0.267	0.0297	0.065*	
H4B	−0.0552	0.3841	0.0482	0.065*	
H4C	−0.1257	0.3698	0.0853	0.065*	
C25	0.07865 (17)	0.2341 (4)	0.39177 (15)	0.0514 (8)	
H25	0.0748	0.1607	0.421	0.062*	
C23	0.09133 (15)	0.4981 (4)	0.36623 (17)	0.0508 (8)	
H23	0.0967	0.5996	0.3766	0.061*	
C15	0.12589 (16)	0.3494 (4)	−0.03822 (14)	0.0448 (7)	
H15	0.0932	0.3439	−0.069	0.054*	
C24	0.08657 (17)	0.3864 (4)	0.40805 (17)	0.0573 (9)	
H24	0.0886	0.4122	0.4477	0.069*	
C14	0.18851 (16)	0.4367 (4)	−0.04522 (14)	0.0455 (7)	
H14	0.1964	0.4871	−0.0806	0.055*	

### Atomic displacement parameters (Å^2^)

**Table:**

	*U*^11^	*U*^22^	*U*^33^	*U*^12^	*U*^13^	*U*^23^
Co1	0.01518 (10)	0.02082 (13)	0.04106 (16)	−0.00018 (11)	−0.00008 (19)	0.00135 (18)
O1W	0.0190 (10)	0.0170 (12)	0.0385 (14)	0.0002 (9)	0	0
O2	0.0257 (7)	0.0211 (9)	0.0618 (14)	0.0010 (7)	−0.0038 (8)	0.0044 (9)
O1	0.0208 (7)	0.0293 (10)	0.0413 (11)	0.0016 (7)	−0.0033 (7)	0.0022 (8)
O4	0.0185 (7)	0.0307 (10)	0.0450 (11)	−0.0010 (6)	−0.0023 (7)	0.0060 (8)
O3	0.0191 (7)	0.0347 (11)	0.0558 (12)	−0.0030 (7)	−0.0059 (8)	0.0147 (9)
N1	0.0189 (9)	0.0282 (12)	0.0429 (13)	−0.0043 (8)	−0.0002 (9)	0.0011 (9)
N4	0.0312 (11)	0.0187 (11)	0.0802 (19)	−0.0033 (9)	0.0069 (12)	−0.0051 (12)
N3	0.0253 (9)	0.0194 (10)	0.0506 (13)	0.0005 (9)	0.0034 (10)	−0.0016 (9)
N2	0.0161 (8)	0.0391 (13)	0.0486 (14)	−0.0069 (8)	0.0019 (9)	0.0006 (11)
C27	0.0199 (9)	0.0234 (13)	0.0562 (17)	0.0021 (10)	−0.0002 (11)	−0.0076 (11)
C26	0.0376 (13)	0.0283 (14)	0.0508 (17)	0.0062 (11)	−0.0028 (12)	−0.0050 (12)
C17	0.0214 (10)	0.0212 (12)	0.0377 (14)	0.0021 (9)	0.0032 (10)	−0.0046 (10)
C22	0.0231 (11)	0.0221 (13)	0.071 (2)	0.0000 (10)	0.0002 (12)	−0.0106 (13)
C12	0.0214 (10)	0.0302 (15)	0.0380 (14)	−0.0002 (9)	0.0044 (9)	−0.0027 (11)
C21	0.0317 (12)	0.0214 (12)	0.0640 (19)	−0.0009 (11)	0.0095 (13)	0.0013 (13)
C11	0.0202 (10)	0.0376 (14)	0.0410 (15)	−0.0032 (10)	−0.0018 (10)	0.0031 (12)
C3	0.0197 (10)	0.0299 (14)	0.0364 (14)	0.0046 (9)	0.0000 (10)	−0.0001 (11)
C13	0.0354 (13)	0.0378 (16)	0.0496 (18)	−0.0009 (12)	0.0130 (12)	0.0006 (13)
C16	0.0276 (11)	0.0312 (15)	0.0462 (17)	0.0012 (11)	−0.0038 (11)	−0.0101 (13)
C2	0.0308 (13)	0.055 (2)	0.0460 (18)	0.0090 (12)	−0.0084 (12)	0.0021 (15)
C1	0.0240 (10)	0.0215 (13)	0.0404 (14)	0.0048 (9)	−0.0031 (10)	−0.0063 (11)
C4	0.0350 (14)	0.0406 (17)	0.0545 (19)	−0.0001 (12)	−0.0108 (14)	0.0165 (15)
C25	0.0548 (18)	0.0489 (19)	0.0504 (18)	0.0122 (15)	−0.0070 (16)	−0.0068 (15)
C23	0.0376 (14)	0.0388 (17)	0.076 (2)	0.0034 (13)	−0.0068 (15)	−0.0244 (18)
C15	0.0474 (15)	0.0504 (19)	0.0367 (16)	0.0072 (14)	−0.0048 (12)	−0.0087 (14)
C24	0.0567 (18)	0.055 (2)	0.060 (2)	0.0147 (16)	−0.0184 (18)	−0.0205 (19)
C14	0.0523 (16)	0.0474 (18)	0.0367 (16)	0.0046 (14)	0.0084 (13)	0.0004 (14)

### Geometric parameters (Å, °)

**Table:**

Co1—O3	2.0495 (18)	C17—C16	1.394 (3)
Co1—O4^i^	2.1044 (19)	C17—C12	1.399 (3)
Co1—O1	2.1141 (17)	C22—C23	1.383 (5)
Co1—O1W	2.1315 (15)	C12—C13	1.389 (4)
Co1—N1	2.139 (2)	C21—H21	0.93
Co1—N3	2.147 (2)	C11—H11	0.93
O1W—Co1^i^	2.1315 (15)	C3—C4	1.501 (4)
O1W—H1W	0.97	C13—C14	1.379 (4)
O2—C1	1.269 (3)	C13—H13	0.93
O1—C1	1.250 (3)	C16—C15	1.379 (4)
O4—C3	1.267 (3)	C16—H16	0.93
O4—Co1^i^	2.1044 (19)	C2—C1	1.508 (3)
O3—C3	1.252 (3)	C2—H2A	0.96
N1—C11	1.314 (3)	C2—H2B	0.96
N1—C17	1.385 (3)	C2—H2C	0.96
N4—C21	1.342 (4)	C4—H4A	0.96
N4—C22	1.375 (4)	C4—H4B	0.96
N4—H22	0.86	C4—H4C	0.96
N3—C21	1.319 (4)	C25—C24	1.400 (5)
N3—C27	1.397 (4)	C25—H25	0.93
N2—C11	1.346 (3)	C23—C24	1.371 (5)
N2—C12	1.371 (4)	C23—H23	0.93
N2—H12	0.86	C15—C14	1.408 (4)
C27—C26	1.391 (4)	C15—H15	0.93
C27—C22	1.402 (4)	C24—H24	0.93
C26—C25	1.384 (4)	C14—H14	0.93
C26—H26	0.93		
			
O3—Co1—O4^i^	97.48 (8)	C13—C12—C17	123.2 (3)
O3—Co1—O1	174.69 (7)	N3—C21—N4	113.3 (3)
O4^i^—Co1—O1	86.88 (7)	N3—C21—H21	123.3
O3—Co1—O1W	90.09 (6)	N4—C21—H21	123.3
O4^i^—Co1—O1W	90.78 (7)	N1—C11—N2	113.1 (2)
O1—Co1—O1W	92.89 (6)	N1—C11—H11	123.4
O3—Co1—N1	88.66 (8)	N2—C11—H11	123.4
O4^i^—Co1—N1	87.79 (8)	O3—C3—O4	125.8 (2)
O1—Co1—N1	88.48 (7)	O3—C3—C4	117.0 (2)
O1W—Co1—N1	177.97 (9)	O4—C3—C4	117.2 (2)
O3—Co1—N3	88.14 (8)	C14—C13—C12	116.4 (3)
O4^i^—Co1—N3	174.38 (8)	C14—C13—H13	121.8
O1—Co1—N3	87.50 (8)	C12—C13—H13	121.8
O1W—Co1—N3	89.38 (8)	C15—C16—C17	117.9 (3)
N1—Co1—N3	92.19 (8)	C15—C16—H16	121.1
Co1^i^—O1W—Co1	116.98 (13)	C17—C16—H16	121.1
Co1^i^—O1W—H1W	108.1	C1—C2—H2A	109.5
Co1—O1W—H1W	108.1	C1—C2—H2B	109.5
C1—O1—Co1	126.63 (14)	H2A—C2—H2B	109.5
C3—O4—Co1^i^	136.38 (16)	C1—C2—H2C	109.5
C3—O3—Co1	136.17 (18)	H2A—C2—H2C	109.5
C11—N1—C17	105.1 (2)	H2B—C2—H2C	109.5
C11—N1—Co1	120.94 (18)	O1—C1—O2	124.4 (2)
C17—N1—Co1	132.86 (15)	O1—C1—C2	118.4 (2)
C21—N4—C22	107.3 (2)	O2—C1—C2	117.2 (2)
C21—N4—H22	126.3	C3—C4—H4A	109.5
C22—N4—H22	126.3	C3—C4—H4B	109.5
C21—N3—C27	104.9 (2)	H4A—C4—H4B	109.5
C21—N3—Co1	121.2 (2)	C3—C4—H4C	109.5
C27—N3—Co1	133.63 (17)	H4A—C4—H4C	109.5
C11—N2—C12	107.17 (19)	H4B—C4—H4C	109.5
C11—N2—H12	126.4	C26—C25—C24	121.9 (4)
C12—N2—H12	126.4	C26—C25—H25	119
C26—C27—N3	131.9 (2)	C24—C25—H25	119
C26—C27—C22	119.2 (3)	C24—C23—C22	117.3 (3)
N3—C27—C22	108.8 (3)	C24—C23—H23	121.4
C25—C26—C27	117.8 (3)	C22—C23—H23	121.4
C25—C26—H26	121.1	C16—C15—C14	121.6 (3)
C27—C26—H26	121.1	C16—C15—H15	119.2
N1—C17—C16	131.3 (2)	C14—C15—H15	119.2
N1—C17—C12	109.1 (2)	C23—C24—C25	120.8 (3)
C16—C17—C12	119.6 (3)	C23—C24—H24	119.6
N4—C22—C23	131.5 (3)	C25—C24—H24	119.6
N4—C22—C27	105.6 (3)	C13—C14—C15	121.3 (3)
C23—C22—C27	122.9 (3)	C13—C14—H14	119.3
N2—C12—C13	131.3 (2)	C15—C14—H14	119.3
N2—C12—C17	105.5 (2)		

Symmetry codes: (i) −*x*, −*y*, *z*.

### Hydrogen-bond geometry (Å, °)

**Table:**

*D*—H···*A*	*D*—H	H···*A*	*D*···*A*	*D*—H···*A*
O1W—H1W···O2	0.97	1.71	2.591 (2)	149
N4—H22···O2^ii^	0.86	1.87	2.717 (3)	167
N2—H12···O4^iii^	0.86	2.00	2.812 (2)	157

Symmetry codes: (ii) *x*, *y*+1, *z*; (iii) *x*+1/2, −*y*+1/2, *z*.
